# Sudden Onset of Coma and Fulminant Progression to Brain Death in a 48-Year-Old Male With Cerebral Malaria

**DOI:** 10.1155/2024/4621985

**Published:** 2024-09-30

**Authors:** Marina Costa, Cristiana Barbosa, Mauro Pereira, Luís Ribeiro, Pedro Silveira

**Affiliations:** Department of Intensive Care Medicine Hospital de Braga, Braga, Portugal

**Keywords:** brain death, case report, cerebral malaria, coma, *Plasmodium falciparum*

## Abstract

Cerebral malaria is the most severe complication of *Plasmodium falciparum* infection. Left untreated, it is universally fatal. Coma is the clinical hallmark, emerging between the first and third days of fever. Adults typically present with mild cerebral edema, usually with a more favorable prognosis compared to the pediatric population. We present a case of a 48-year-old man with a recent travel to Angola who presented comatose on the second day of a febrile illness with clinical signs of cerebral herniation and diffuse cerebral edema and cerebellar tonsil ectopia on cranioencephalic computed tomography. He had a missed diagnosis on a first visit to the emergency department 2 days prior. The diagnosis of cerebral malaria was confirmed after the identification of the parasite in peripheral blood. He was admitted to an intensive care unit; however, progression to brain death was inevitable within a few hours. Malaria affects 5% of the world's population. In Portugal, it has an incidence of 0.01 in every 1000 inhabitants, and all cases are imported. Despite its rarity in a nonendemic country, its severity alerts to the consideration of this syndrome in the etiologic workup of coma. The early recognition of the diagnosis is of major importance for the establishment of definitive treatment, as its timely administration has a crucial impact on the outcome.

## 1. Introduction

Cerebral malaria (CM) is the most severe neurologic complication of the infection by *Plasmodium falciparum*, and it is almost universally fatal without treatment. Coma is the clinical hallmark, emerging between the first and third days of fever. A mortality of 15%–20% is observed with timely and appropriate treatment with effective antimalarials [1]. In malaria-endemic countries, mortality in children is most frequently related to brain herniation secondary to diffuse cerebral edema, whereas the adult patient usually presents with mild cerebral edema, rarely leading to coma or death [2]. The early consideration of CM in patients presenting with neurological symptoms and recent travel history to malaria-endemic countries, followed by early administration of antimalarials, may have a critical impact on prognosis [3].

We report a case of severe *P. falciparum* imported malaria, who had a missed diagnosis on a first emergency department (ED) visit, presenting diffuse cerebral edema with progression to brain death a few hours after coma onset, on the second day of fever.

## 2. Case Description

A 48-year-old Portuguese male, with a working contract in Angola and regular temporary visits, with no prior medical history, was brought by the prehospital emergency team for coma. The team described, upon arrival on site, a Glasgow Coma Scale of 3, no pupillary abnormalities, bradypnea, and hypertension (240/110 mmHg). After orotraqueal intubation, mannitol administration ensued due to fixed bilateral mydriasis. Recent travel to Angola, for 3 weeks, was referred by the family, and no chemoprophylaxis was taken. In addition, they mentioned a visit to the ED 2 days prior with complaints of fever and headache, having been discharged with antipyretics, without pondering for malaria testing. Thereafter, he developed refractory fever, new-onset dysarthria, and vomiting, with progression to unresponsiveness, prompting a call to the emergency service.

**Figure 1:**
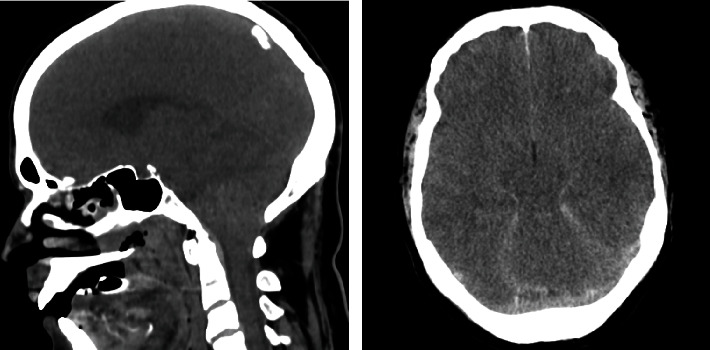
Sagittal and axial views of cranial computed tomography imaging showing diffuse cerebral edema, with loss of grey–white matter differentiation, obliteration of cisterns and sulcal spaces, and cerebellar tonsil herniation.

On arrival at the emergency room, the patient presented in a coma with fixed mydriasis, absence of corneal or oculocephalic reflexes, tachycardia (120 bpm), and normotension (100/50 mmHg). Initial laboratory studies showed mixed acidemia and hyperlactatemia of 4.7 mmol/L, anemia of 12.5 g/dL, thrombocytopenia of 21,000/*μ*L, prolonged prothrombin time of 16.6 s, hyperbilirubinemia of 4.5 mg/dL, and C-reactive protein of 165 mg/L, with slight elevation of plasma creatinine and myocardial necrosis markers. Cranial computed tomography revealed signs of diffuse cerebral edema with cerebellar tonsillar ectopia (Figure 1). The diagnosis of CM was established after the detection of *P. falciparum* trophozoites in blood smears, with a parasitemia of 7%.

The patient was admitted to the intensive care unit and started treatment with quinine and supportive care. The following hours were marked by signs of progressing brainstem distress. On the second day of admission, brain death was diagnosed by neurological criteria.

## 3. Discussion

Here, we report a rare case of an adult patient with imported CM with an atypically fulminant and fatal evolution in Portugal. Some of its interesting distinctive features are the sudden emergence of coma, instead of the usually described gradual evolution to a comatose state; the rapid development of brain swelling, 2 h after coma onset; and the brainstem distress, rarely described in adult patients. Another important highlight of this case is the diagnostic delay, unrecognized on his first ED visit, probably due to a lapse of communication about the patient's travel history, leading to a deferral on treatment commencement.

Cerebral edema in CM is usually more severe in children, whereas in adults, it is mostly found as part of a multiorgan failure syndrome [4, 5]. Typically, in adults, coma evolves gradually, lasting for 48 h, and brainstem compromise is rare in comparison to the pediatric population [6]. In the majority of survivors, no major neurological deficit is detectable, suggesting that the processes leading to coma may be rapidly and completely reversible. The mechanisms of neural injury in CM are poorly understood. Investigators suggest that parasite sequestration in cerebral microvasculature might explain cerebral ischemia and endothelial dysfunction with blood–brain barrier disruption promoting astroglial activation by neurotoxins [3, 6–9]. Postmortem studies suggest that cerebral edema might not be directly related to coma as it was observed in patients with cerebral and non-CM [10].

The case described points to the serious consequences possibly associated with globalization and increased air connectivity between endemic and nonendemic countries. Malaria affects 5% of the world's population. Worldwide, there were 249 million cases of malaria during 2022 [11]. In Portugal, an incidence of 0.01 cases per 1000 inhabitants was reported in 2018, and all cases were imported [12, 13]. European Union countries reporting a higher number of malaria cases have historical, economic, linguistic, and cultural ties with endemic areas, particularly in Africa and the United States. This highlights the importance of travel medicine and its work on pretravel counseling and prevention measures. Chemoprophylaxis is independently associated with a decreased risk of dying from the disease in nonimmune patients, and it appears that the severity of illness is lower among patients who take chemoprophylaxis compared to patients who do not [14]. In this case, not taking chemoprophylaxis might have contributed to a more severe progression of the disease.

This case also highlights the importance of conducting a thorough and directed medical history due to its potential impact on the prognosis of a patient presenting in a coma. The fact that the patient was discharged home on his first ED visit shows a critical mismanagement that may have contributed to the rapid progression of the disease, highlighting a serious lapse in clinical judgment by not considering malaria in the differential diagnosis. Moreover, it highlights the importance of having ED medical teams up to date on the clinical management of these patients. Artesunate is recommended as the treatment of choice for malaria worldwide, following its significant impact on survival in severe malaria patients compared to quinine [15]. Additionally, there is evidence of potential harm in administering mannitol to patients with CM, as it prolongs coma duration, although in this case, the diagnosis was not established by the time of its administration [16]. Despite its rarity in a nonendemic country, its severity calls for consideration of CM in the etiological study of acute-onset coma, particularly in patients who have recently traveled to malaria-endemic regions.

## 4. Conclusion

CM is a severe complication of *P. falciparum* infection. The case described depicts an uncommon fulminant evolution of CM, progressing to brain death due to brain swelling. It highlights the importance of considering the diagnosis of malaria in a patient with fever and recent travel to an endemic region, as well as CM if coma is also present. A lapse in early diagnosis can incur serious hazards to the patient. This case alerts for the importance of educating ED doctors and students on the potential impact of approaching travel history on a patient's first visit to the ED with a fever.

## Data Availability

The data that support the findings of this case report are available on request from the corresponding author, Marina Costa, upon reasonable request. The data are not publicly available due to the sensitive nature of the information.

## References

[1] (2014). Severe malaria. *Tropical Medicine & International Health*.

[2] Maude R. J., Barkhof F., Hassan M. U. (2014). Magnetic resonance imaging of the brain in adults with severe falciparum malaria. *Malaria Journal*.

[3] Idro R., Marsh K., John C. C., Newton C. R. (2010). Cerebral malaria: mechanisms of brain injury and strategies for improved neurocognitive outcome. *Pediatric Research*.

[4] Sanni L. A. (2001). The role of cerebral oedema in the pathogenesis of cerebral malaria. *Redox Report*.

[5] Dondorp A. M., Lee S. J., Faiz M. A. (2008). The relationship between age and the manifestations of and mortality associated with severe malaria. *Clinical Infectious Diseases*.

[6] Idro R., Jenkins N. E., Newton C. R. (2005). Pathogenesis, clinical features, and neurological outcome of cerebral malaria. *The Lancet Neurology*.

[7] MacPherson G. G., Warrell M. J., White N. J., Looareesuwan S., Warrell D. A. (1985). Human cerebral malaria. A quantitative ultrastructural analysis of parasitized erythrocyte sequestration. *The American Journal of Pathology*.

[8] Sahu P. K., Hoffmann A., Majhi M. (2021). Brain magnetic resonance imaging reveals different courses of disease in pediatric and adult cerebral malaria. *Clinical Infectious Diseases*.

[9] Lucas S. B., Hounnou A., Bell J. (1996). Severe cerebral swelling is not observed in children dying with malaria. *The Quarterly Journal of Medicine*.

[10] Medana I. M., Day N. P. J., Sachanonta N. (2011). Coma in fatal adult human malaria is not caused by cerebral oedema. *Malaria Journal*.

[11] World Health Organization (2022). *World malaria report 2022*.

[12] Instituto nacional de estatística (2018). Taxa de incidência da malária por 1000 habitantes. https://www.ine.pt/xportal/xmain?xpid=INE%26xpgid=ine_indicadores%26indOcorrCod=0009672%26contexto=bd%26selTab=tab2.

[13] Reis T., Martins S., Ferreira I., Vilares A., Gargate M. J. (2021). Malária: estudo retrospetivo de casos clínicos suspeitos de infeção por Plasmodium sp.(2010-2020). *Boletim Epidemiológico Observações*.

[14] Krause G., Schöneberg I., Altmann D., Stark K. (2006). Chemoprophylaxis and malaria death rates. *Emerging Infectious Diseases*.

[15] Dondorp A. M., Fanello C. I., Hendriksen I. C. (2010). Artesunate versus quinine in the treatment of severe falciparum malaria in African children (AQUAMAT): an open-label, randomised trial. *The Lancet*.

[16] Mohanty S., Mishra S. K., Patnaik R., Dutt A. K., Pradhan S., Das B. (2011). Brain swelling and mannitol therapy in adult cerebral malaria: a randomized trial. *Clinical Infectious Diseases*.

